# Aetiology and outcomes for dialysis-dependent acute kidney injury patients on the ICU

**DOI:** 10.1186/cc12366

**Published:** 2013-03-19

**Authors:** M Hameed, P Carmichael

**Affiliations:** 1 Salford Royal NHS Foundation Trust, Salford, UK; 2 The Royal Wolverhampton Hopsitals NHS Trust, Wolverhampton, UK

## Introduction

AKI is a common occurrence in sick hospitalized patients, in particular those admitted to intensive care. Published data suggest that 4 to 5% of all critically ill patients develop severe AKI and require initiation of renal replacement therapy (RRT) [1,2]. Such patients have high mortality rates often exceeding 60% [2]. We aimed to review the outcomes of patients admitted to the ICU and required renal replacement therapy for AKI. We examined whether aetiology of AKI, comorbidity burden, hospital length of stay and treatment in ICU had any significant association with survival in the study cohort.

## Methods

During 2009, 56 patients were identified to have received RRT with AKI who were admitted to the ICU at the Royal Wolverhampton Hospitals NHS Trust. Computerised and paper-based case records were examined for these patients to collect the data. AKIN classification was used to classify the severity of AKI.

## Results

Median age at admission was 66 years (27 to 85) with 29 males and 27 females. Thirty-one (55.4%) patients had sepsis and 20 (35.7%) patients had ATN as the main cause of AKI. Thirty-two patients (57%) had three organ failures at the time of commencement of RRT. Forty-six patients (82.1%) received haemofiltration only. Thirty-two (57%) patients died, with more than 80% of these occurring in the ITU. There was no significant difference in survival when compared with duration of haemofiltration, length of stay, number of organs failed and number of comorbidities. However, significantly more patients that died had AKI due to sepsis (*P *= 0.003) or if they received mechanical ventilation (*P *= 0.48) or inotropes (0.04). Of the 27 patients who survived until discharge from hospital, 18 (66.7%) had normal renal function, eight (29.6%) had AKIN stage I and only one patient required maintenance haemodialysis.

## Conclusion

Individuals who develop dialysis-dependent AKI in the ICU setting in general terms either die or recover. Sepsis is the most common association with death. The need for mechanical ventilation and inotropic therapy are both associated with increased incidence of death.

## References

[B1] Metnitz PG Effect of acute renal failure requiring renal replacement therapy on outcome in critically ill patients Crit Care Med 2002 30 2051 2058 10.1097/00003246-200209000-00016 12352040

[B2] Uchino S Acute renal failure in critically ill patients: a multinational, multicenter study JAMA 2005 294 813 818 10.1001/jama.294.7.813 16106006

